# A Non-Contact Measuring System for In-Situ Surface Characterization Based on Laser Confocal Microscopy

**DOI:** 10.3390/s18082657

**Published:** 2018-08-13

**Authors:** Shaowei Fu, Fang Cheng, Tegoeh Tjahjowidodo, Yu Zhou, David Butler

**Affiliations:** 1 Advanced Remanufacturing and Technology Centre (Agency for Science, Technology and Research), Singapore 637143, Singapore; FUSH0009@e.ntu.edu.sg (S.F.); ttegoeh@ntu.edu.sg (T.T.); zhouy@artc.a-star.edu.sg (Y.Z.); 2 School of Mechanical and Aerospace Engineering, Nanyang Technological University, Singapore 637798, Singapore; 3 Design, Manufacture and Engineering Management Department, University of Strathclyde, Glasgow G11XQ, UK; david.butler@strath.ac.uk

**Keywords:** surface roughness, non-contact, in-situ measurement, error correction

## Abstract

The characterization of surface topographic features on a component is typically quantified using two-dimensional roughness descriptors which are captured by off-line desktop instruments. Ideally any measurement system should be integrated into the manufacturing process to provide in-situ measurement and real-time feedback. A non-contact in-situ surface topography measuring system is proposed in this paper. The proposed system utilizes a laser confocal sensor in both lateral and vertical scanning modes to measure the height of the target features. The roughness parameters are calculated in the developed data processing software according to ISO 4287. To reduce the inherent disadvantage of confocal microscopy, e.g., scattering noise at steep angles and background noise from specular reflection from the optical elements, the developed system has been calibrated and a linear correction factor has been applied in this study. A particular challenge identified for this work is the in-situ measurement of features generated by a robotized surface finishing system. The proposed system was integrated onto a robotic arm with the measuring distance and angle adjusted during measurement based on a CAD model of the component in question. Experimental data confirms the capability of this system to measure the surface roughness within the *Ra* range of 0.2–7 μm (bandwidth *λ_c_/λ_s_* of 300), with a relative accuracy of 5%.

## 1. Introduction

Surface topography measurements play an important role in product quality assessment for manufacturing process evaluation. Surface roughness, calculated from surface topographic information, is widely used for surface characterization. In most applications, compared to contour measurements, surface topography measurements require higher resolution and accuracy [1,2], and the process typically relies on laboratory equipment. This makes an in-situ surface topography measurement and roughness analysis [3,4] very challenging as it has to meet the compactness and robustness requirements, especially in a relatively harsh manufacturing environment.

The conventional approach to measuring the surface topography is to drag a physical stylus across the surface in a raster motion in order to capture the surface height deviations [5]. Contact measurement is limited to a relatively low measurement speed (<1 mm/s) in order to avoid the stylus jumping [6], data is captured at discrete intervals along the surface. Non-contact systems based on optical techniques, such as confocal microscopy [6,7], focus variation microscopy [8] and coherence scanning interferometry [9,10], are commercially available for high accuracy measurements. These optical systems are able to achieve lateral resolution of around 0.5 μm and vertical resolution down to sub-nanometer range [11]. However, due to the complex alignment procedures and limitation of the measurement space, most optical profilers come in desktop form, which make them impractical for in-situ measurement. Chromatic confocal sensors [12,13], which are based on the reflected light spectrum, are also applicable for high-accuracy dimensional measurement. Its highest spatial resolution of 5 μm [14], however, limits its application for surface roughness measurement. Recently, a surface topography measurement system was proposed using a chromatic confocal sensor [15]. However, no experimental data was provided for smooth surfaces with *Ra* less than 1 µm. Taking in consideration the resolution, system robustness and spot size, single-point confocal technology could provide a potential solution for in-situ surface roughness measurement [16].

In this paper, we propose a non-contact in-situ surface topography measuring system and the paper is organized as follows: Section 2 compares stylus-based and confocal-based methods for surface roughness measurement. Section 3 presents the proposed system configuration and validation for surface roughness measurement. Section 4 analyses the experimental work and measurement data. Conclusions and proposed future work are presented in the final section.

## 2. Stylus and Confocal Methods for Surface Profile Measurement

In this study, the surface profile measurement using the proposed confocal microscopy-based system will be benchmarked against a high resolution stylus profilometer using a phase grating interferometer (PGI) transducer. This section discusses both the systems in brief.

### 2.1. Stylus Profilometry

Conventionally, the stylus profilometer serves as a standard method for surface roughness measurement [17,18]. The stylus tip physically senses the sample surface and traverses across the surface at a constant speed with a static measuring force of 0.75 mN according to ISO 3274 [5]. The vertical displacement of the stylus due to the surface profile is then converted to either a digital signal by an optical sensing transducer, or an analog signal by a linear variable differential transformer (LVDT) and subsequently digitized [19]. The signal is then processed and analyzed to generate the surface profile.

In this study, a commercial stylus profilometer that utilizes a phase grating interferometer (PGI) transducer [20] capable of achieving sub-nanometer vertical resolution, is used as a benchmark. In the system, a laser beam is directed onto a convex diffraction grating at normal incidence and generates two first order diffraction beams. The vertical movement of the stylus arm on the pivot is converted to a rotation of the diffraction grating and generate a frequency shift of the diffracted beams. Finally, the position of the stylus can be calculated from the phase of the interference signal generated by superimposing the diffracted beams [17,21,22].

### 2.2. Confocal Microscopy

**Figure 1:**
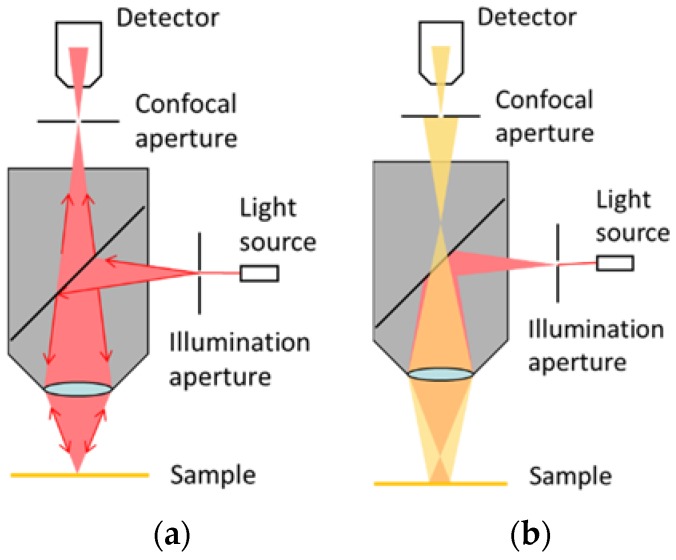
Structure of single-point confocal system. (**a**) Target point is on focus; (**b**) Target point is out of focus.

Figure 1 illustrates the typical optical structure of a single-point confocal system. Most reflected light will pass through the pinhole only when the target point is on the focal plane. In the range of confocal system’s Depth of Field (DOF), the reflected light intensity detected by the photodiode forms a Depth Response Curve (DRC). The peak point of the DRC detected by the photodiode indicates the focus plane of the target point on the measured surface [23,24]. With a high-resolution encoder of the confocal system, the height of the target point on the surface can be measured. Thus through the recording of the heights, a surface profile can be obtained and roughness derived [25,26].

Apart from the pinhole scanning technique, some confocal microscopes use the slit scanning method. The slit scanning method has an advantage of increasing the signal intensity and speed, but its vertical resolution is significantly deteriorated by the lateral cross-talk [13]. Another critical consideration for surface measurement is the lateral resolution. Compared to white light, as a point light source, laser can achieve a smaller spot size. In addition, laser has more concentrated power than that of the white light. This allows higher light intensity levels at the focal point [27].

With respect to the above consideration of vertical and lateral resolution, in this study a single-point laser confocal sensor, Keyence LT-9010M (KEYENCE, Osaka, Japan), is employed for developing an in-situ roughness measurement system. The sensor utilizes a red semiconductor laser with a wavelength of 655 nm. The laser beam spot diameter is 2 µm and the vertical resolution of the laser confocal sensor is 0.1 µm. In addition to the basic optical system, the laser confocal sensor has two embedded scanning mechanisms, which determine a vertical measurement range of 0.6 mm and a lateral scanning length of 1.1 mm.

**Figure 2:**
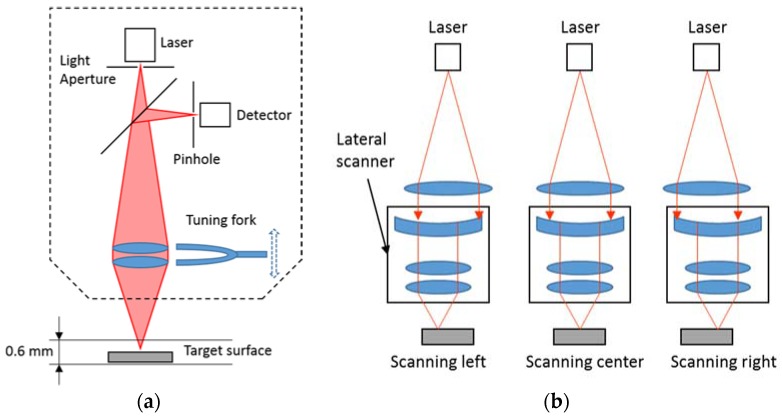
Working principle of the laser confocal sensor. (**a**) Vertical scanning mechanism; (**b**) Lateral scanning mechanism.

Figure 2 shows the working principle of the laser confocal sensor. By vertical scanning of the objective lens using a tuning fork, the detector will receive the highest light intensity when the target surface is located at the focal distance. The internal sensor attached to the tuning fork determines the target height by measuring the position of the turning fork. The embedded lateral scanner helps to achieve a measuring length of 1.1 mm by using a high accuracy oscillating mechanism. A 2D surface profile, in the form of (X, Z) data points, is sent to the control station via serial communication and then processed in real-time by an algorithm to compute the surface roughness. The sampling frequency of the laser confocal sensor is able to achieve 1.5 kHz, which is suitable for surface profile measurement.

### 2.3. Comparison of Stylus and Laser Confocal Measurement Methods

Contact surface measurement using a stylus has been the standard method employed by academia and industry for over 70 years. The stylus profilometer can achieve a vertical resolution down to 0.1 nm using a phase grating interferometer (PGI) transducer or linear variable differential transformer (LVDT). The spatial resolution is limited by the stylus tip size and shape, which can be as small as 0.1 µm [11]. However, the main drawback of the stylus measurement method is attributed to the damage to the measured surface due to the applied contact force [17]. In addition, the stylus tip and the PGI transducer are often too sensitive to be used in a manufacturing environment for in-situ measurement [18].

To overcome the disadvantages of the stylus method, the laser confocal technique is considered to be a potential alternative for non-contact and non-destructive surface measurement. The laser confocal sensor used in this experimental study is integrated with vertical and lateral scanning mechanisms. This technique solves the problem of the autofocus systems which need to move the scanning unit in every sectioning step [28]. Compared to typical stylus measurement speed of 1 mm/s, the measurement speed of the developed laser confocal system can reach up to 3 mm/s, which allows in-situ surface roughness measurement.

## 3. System Configuration and Validation

### 3.1. Surface Roughness Calculation

In general, any surface profile comprises of roughness, waviness and form features. Roughness is an irregularity as a result of any production processes such as tearing, cutting and surface fatigue. Waviness is a periodic texture, usually caused by vibration, chatter or machine deflections. Form often results from inaccuracies of the machine elements such as elastic deformations, linear guide errors and long-term thermal effects.

In order to obtain the roughness profile, the surface form needs to be separated from the surface profile. To eliminate the surface form from the measurement result, best-fit least-squares methods are recommended in ISO 4287 [25]. A second order polynomial fitting method using least squares algorithm was introduced for illustration purpose. The surface roughness standards set (FLEXBAR SKU-16008, Flexbar, Islandia, NY, USA) used in this study are machined by grinding, turning and milling processes. The form errors introduced by these machining processes are relatively simple, such as lines and curvatures, which make a second order polynomial fitting suitable for form error removal.

To separate short wave components such as micro-fracture marks and a waviness profile from the roughness profile, a Gaussian profile filter has been introduced following ISO 16610-21 [29]. The Gaussian profile filter is a phase correct filter that does not result in phase shift and asymmetrical profile distortion [30]. The weighting function for the Gaussian profile filter is given by Equation (1):

(1) $$\;s\left(x\right)=\frac{1}{\alpha \times \lambda }\times exp\left[-\pi {\left(\frac{x}{\alpha \times \lambda }\right)}^{2}\right]\;$$

where *x* is the distance from the center of the weighting function; α equals to $\sqrt{\ln2/\pi }$ to provide 50% transmission characteristic of the Gaussian profile filter at the cut-off wavelength  λ. The long cut-off wavelength $\lambda_{c}$ is determined based on ISO 4288 [31] to separate the waviness profile. The short cut-off wavelength $\lambda_{s}$ defines the intersection between roughness and even shorter wave components. The cut-off wavelength ratio $\lambda_{c}/\lambda_{s}$ should be determined based on ISO 3274 [5]. The primary surface profile $Z_{P}\left(x\right)$ can be obtained by applying short wavelength filter of $\lambda_{s}$.

The waviness profile $Z_{W}\left(x\right)$ is the discrete convolution of the primary profile $Z_{P}\left(x\right)$ and weighting function s(x) given by Equation (2):

(2) $$\;Z_{W}\left(x\right)=\sum_{i=x-L_{c}\lambda_{c}}^{x+L_{c}\lambda_{c}}Z_{P}\left(i\right)s\left(x-i\right)\;$$

where, $L_{c}$ is the truncation constant of the weighting function. For general use following ISO 16610-21 [29], $L_{c}$ equals to 0.5 and results in a 0.76% implementation error.

The roughness profile $Z_{R}\left(x\right)$ is the deduction between the leveled surface profile $Z_{L}\left(x\right)$ and the waviness profile $Z_{W}\left(x\right)$ given by Equation (3):

(3) $$\;Z_{R}\left(x\right)=Z_{L}\left(x\right)-Z_{W}\left(x\right)\;$$

In this study, the surface roughness parameter used to validate the developed surface measurement system is the arithmetical mean roughness (*Ra*), the most widely used surface texture parameter. The definition of *Ra* parameter following ISO 4287 [32] is expressed by Equation (4):

(4) $$\;Ra=\frac{1}{n}\sum_{i=1}^{n}\left|Z_{R}\left(i\right)\right|\;$$

The surface roughness calculation algorithm presented in Equations (1)–(4) is implemented in the developed data processing software for in-situ surface roughness measurement.

### 3.2. Internal Scanning Performance of the Laser Confocal Sensor

**Figure 3:**
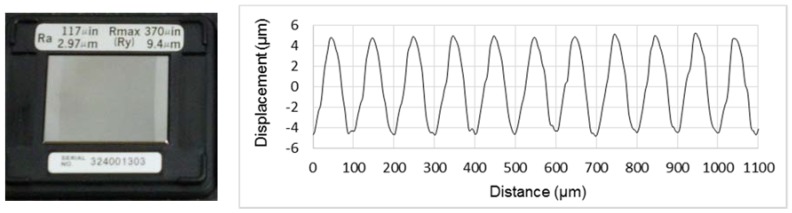
Precision roughness reference specimen and its measured surface profile.

In this study, a precision roughness reference specimen (Mitutoyo 178-602, Mitutoyo Corporation, Kawasaki-shi, Japan) with calibrated *Ra* value of 2.97 μm is used to verify the measurement capability of the laser confocal sensor (LCS). The surface pattern of this reference specimen has a harmonic form and the surface profile measured from the LCS is illustrated in Figure 3. This reference specimen was measured five times on the same spot using the LCS and the *Ra* values listed in Table 1 give a mean value of 3.07 ± 0.05 μm (mean ± 1 std. dev). The measurement results show a good correlation with the stated *Ra* value and demonstrate the feasibility of surface roughness measurement using the LCS.

### 3.3. Extended Measurement Range and Profile Data Stitching Algorithm

The measurement length achieved by the embedded scanner was 1.1 mm, which can only cover one cut-off length of 0.8 mm when the surface *Ra* value is in the range of 0.1–2 μm (following ISO 4288 [31]). However, measurement of five cut-off lengths is the default recommendation according to ISO 4288. In order to expand the lateral measuring range and improve the vertical positioning accuracy of the laser confocal sensor (LCS), a compact XZ-configured 2-axis motorized linear stage (New Focus 9066-XY-PP-M, Newport Corporation, Irvine, CA, USA) is integrated to improve the lateral scanning range. The XZ-configured linear stage has a travel range of 12.7 mm and a minimum incremental motion of 30 nm in both X and Z axis. By accessing the Dynamic Link Library (DLL) in the driver of the XZ-configured linear stage, the in-house developed software is able to set the speed, acceleration and PID control parameters of the linear stage.

The Z-axis linear stage carries the LCS and moves to its working distance of 6 mm from the sample surface. The LCS performs a local scan of 1.1 mm length to measure the surface profile and outputs the surface profile data to the developed data processing software. Then, the X-axis linear stage moves a distance equal to 80 percent of the local scan length in order to overlap 20 percent of the two adjacent surface profiles.

**Figure 4:**
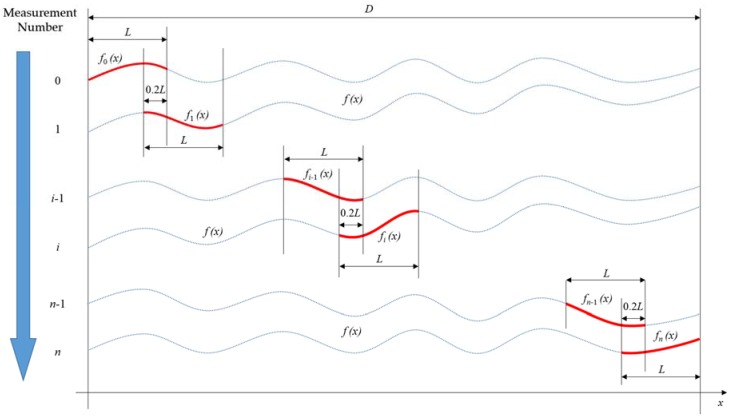
Model of *n*-times of profile data stitching.

Due to the misalignment between the LCS scanning axis and linear stage movement axis, jump errors were observed at the overlapping surface profile. To reduce this misalignment error, a data stitching algorithm was introduced, which was based on the iterative least-square method. Figure 4 shows the model of n-times of surface profile data stitching, where *f*(*x*) and D represent the entire profile to be measured and its length, *f_i_*(*x*) and L represent part of the whole profile to be measured and its length in each measurement, 0.2 L represents a 20 percent overlapping length between two adjacent surface profiles. Several publications [33,34,35] report that a 20 percent overlapping surface will give a good trade-off between having good stitching accuracy and obtaining large measurement range with minimum data sets.

Assuming that the local surface roughness information is consistent in the overlapping area of the two adjacent surface profiles, the mismatch is only caused by slope and offset differences during measurement. The error propagation of the stitching algorithm has been analyzed in [36,37,38] and was shown that the stitching error was in the tens of nanometer level for a range longer than 50 mm stitched length.

In Equation (5), $\Delta f_{i}\left(x\right)$ denotes the difference between $f_{i-1}\left(x\right)$ and $f_{i}\left(x\right)$ within the overlapping section. In Equation (6), $a_{i}$ and $b_{i}$ represent the slope and offset coefficients in the least-square linear regression equation, respectively:

(5) $$\;\Delta f_{i}\left(x\right)=\;f_{i-1}\left(x\right)-f_{i}\left(x\right);\;x\in \left[\;0.8iL,\;0.8iL+0.2L\right]\;$$

(6) $$\;\Delta f_{i}\left(x\right)=\;a_{i}x\;+\;b_{i};\;x\in \left[\;0.8iL,\;0.8iL+0.2L\right]\;$$

In this *n*th iterative stitching algorithm, $f_{i}^{'}\left(x\right)$ corresponds to the shifted profile in the *i*th iteration derived in Equation (7):

(7) $$\;f_{1}^{\prime }\left(x\right)=\;f_{1}\left(x\right)+a_{1}x+b_{1};\;x\in \left[\;0.8L,\;1.8L\right]\;\;\;f_{2}^{\prime }\left(x\right)=\;f_{2}\left(x\right)+a_{2}x+\;b_{2}+a_{1}x+b_{1};\;x\in \left[\;1.6L,\;2.6L\right]\;\;f_{n}^{\prime }\left(x\right)=\;f_{n}\left(x\right)+\sum_{i=1}^{n}(a_{i}x+\;b_{i});\;x\in \left[\;0.8nL,\;0.8nL+L\right]$$

By adding both sides of Equation (7), the whole stitched surface profile f(x) can be written as shown in Equation (8):

(8) $$\;f\left(x\right)=\;f_{0}\left(x\right)+\sum_{i=1}^{n}f_{i}^{\prime }(x)\;$$

**Figure 5:**
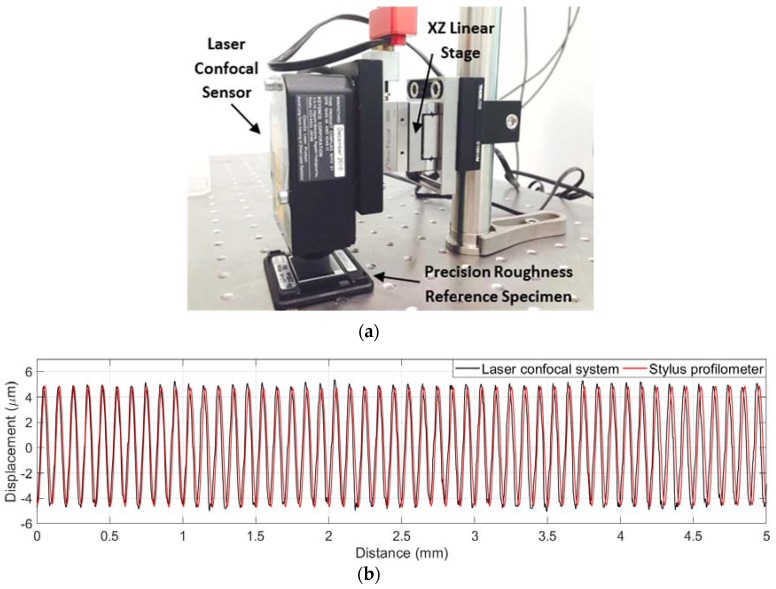

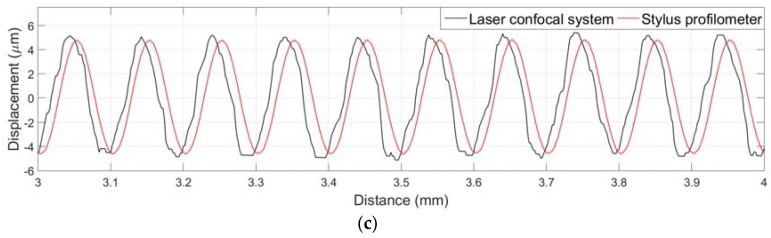
(**a**) Laboratory set-up; (**b**) Extended surface profiles of the precision roughness reference specimen; (**c**) Enlarged partial profiles of the precision roughness reference specimen.

With the integration of the XZ-configured linear stage and data stitching algorithm, the proposed system could achieve a surface profile measurement up to 12.7 mm in length. The laboratory set-up of the proposed system and the precision roughness reference specimen are shown in Figure 5a. To verify the extended data stitching algorithm, the precision roughness reference specimen was measured using the proposed laser confocal system and the Talysurf PGI 800 stylus profilometer (AMETEK Inc., Berwyn, PA, USA) separately. The measured surface profiles are plotted in Figure 5b. As can be seen from this figure, a good match between the laser confocal profile and the stylus profile was observed. 

To compare the discrepancies between the two profiles in Figure 5b, the enlarged partial surface profiles are plotted in Figure 5c. It can be observed that the laser confocal profile has higher peak-to-valley amplitudes compared to that from the stylus profile. It also indicates noise-like spikes at the profile peaks and valleys. In addition, the laser confocal profile is not as symmetric as that from the stylus profile, which is mainly attributed to the light scattering effect at the steep slope section in the surface profile. These observations are consistent with the results from previous studies [11,39,40], which showed that the area of high curvature at surface peaks and valleys may produce severe measurement distortions and noises. 

The Mean Squared Error (MSE) of the five overlapping sections in the stitched laser confocal profile are presented in Table 2. The stitching error in the form of MSE is 0.024 ± 0.010 μm (mean ± std. dev). The stitching error is still acceptable referring to the sample’s *Ra* value of 3 μm.

### 3.4. Step Height Measurement

Step heights of six depth measurement standards (type A1) were measured following the guidelines in ISO 5436-1 [41]. Five measurements have been distributed evenly over each depth measurement standard.

Table 3 summarizes the calculated step heights of six depth measurement standards measured by Talysurf PGI 800 stylus profilometer and the proposed laser confocal system. It can be observed that although all measurement results are in good correlation, the confocal measurement results have slightly higher values compared to the stylus measurement results. This may be due to the measurement distortions [11] and noises at the high curvature surface area which is explained in Section 3.3.

### 3.5. In-Situ Measurement Procedure

The proposed system is also integrated with a 3-stage motion control to minimize vibration caused by the positioning system or scanning mechanism. For in-situ measurement, the motion system, which could be a robot or other motion stages, may cause notable vibration. Lateral scanning is also another source of vibration, which is an important consideration for conventional off-line measurement systems. In this research work, in order to minimize the vibration, the proposed system only actuates the embedded scanning mechanism simultaneously with data collection. The robot arm is used only for approaching and alignment operations, roughly adjusting the measuring distance and angle.

In this process the measurement system is positioned based on the CAD model of the component. The Z-axis linear stage is used to precisely move the laser confocal sensor (LCS) to its working distance of 6 mm from the work piece surface. The X-axis linear stage is used to shift the LCS for every 0.88 mm interval. When placement of the LCS is completed, the motion function of the robot arm and XZ-configured linear stage will be disabled.

**Figure 6:**
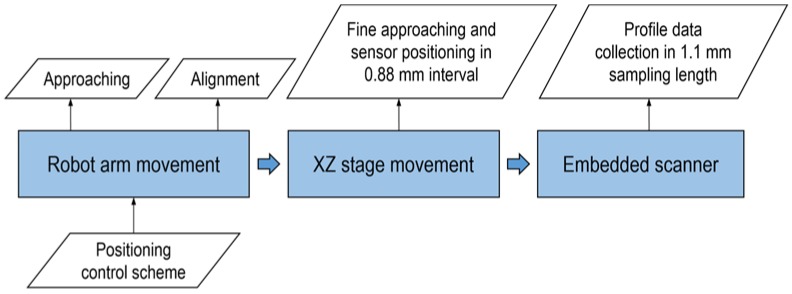
Three-stage movement strategy for surface measurement.

In every positioning interval, surface scanning of 1.1 mm is done by the embedded lateral scanner in the LCS. During the surface data collection, the embedded lateral scanner, where the vibration is negligible, is the only moving unit. Upon the completion of the measurement procedure, the profile data in every interval will be stitched together using the developed algorithm described in Section 3.3. The motion control algorithm for in-situ surface roughness measurement can be summarized as shown in the flowchart in Figure 6.

## 4. Experimental Work

It has been discussed in Section 3.1 that following ISO 4288 [28], surface roughness can be calculated based on the surface profile collected by the developed laser confocal measuring system. In order to determine the accuracy of the proposed confocal system, the Talysurf PGI 800 stylus profilometer is used as a reference. A series of roughness standard data from several samples representing different machining processes with nominal *Ra* value in a range of 0.2–6.3 μm were used for calibration purpose. The error curve of the proposed system and error compensation are presented in this section.

### 4.1. Experimental Setup and Roughness Measurement

**Figure 7:**
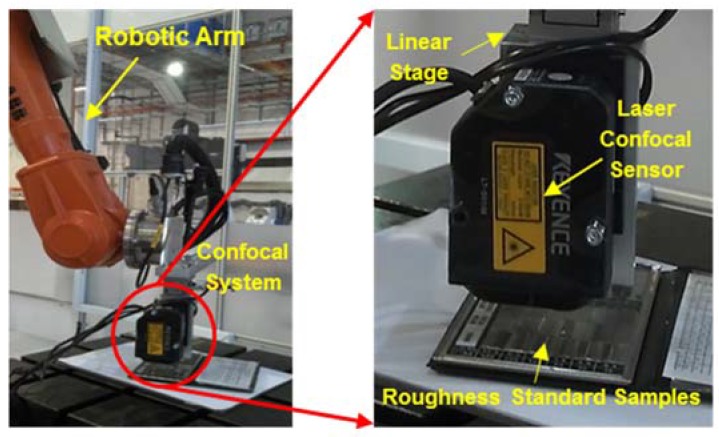
Experimental system setup.

The experimental setup in a robot cell is illustrated in Figure 7. The developed laser confocal measuring system is integrated with an industrial robot (ABB IRB 2400, ABB Group, Zurich, Switzerland) for roughly positioning and alignment. This industrial robot provides a payload of 12 kg and position repeatability of 0.05 mm.

**Figure 8:**
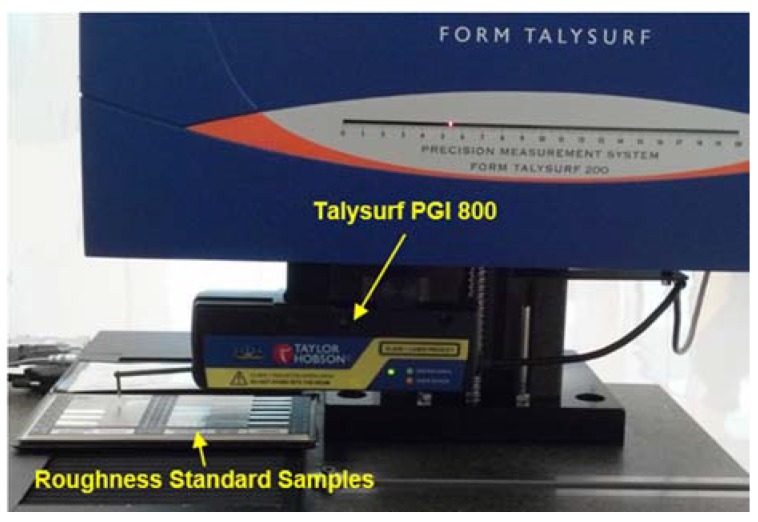
Schematic of Talysurf PGI 800 during measurement of roughness standard samples.

The Talysurf PGI 800 stylus profilometer shown in Figure 8 is used as a reference instrument. The stylus profilometer is a high accuracy instrument and widely used as a standard instrument for surface roughness measurement. It employs a phase grating interferometer to trace the diamond tip stylus that is in contact to the target surface to achieve nanometer resolution.

The roughness standard samples have been repeatedly measured five times using both Talysurf PGI 800 and the proposed laser confocal measuring system. By comparing the averaged roughness measurement results, the accuracy and measuring range of the proposed laser confocal system is assessed.

### 4.2. Data Analysis

**Figure 9:**
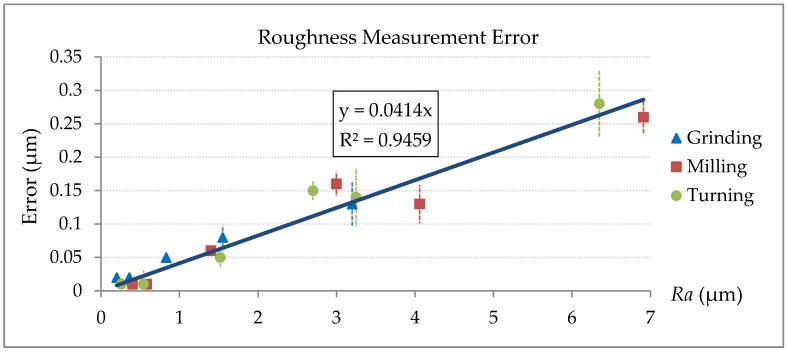
Error curve of different machining surface.

Table 4 shows the roughness standard samples measurement results of the stylus profilometer Taylor Hobson PGI 800 and the proposed laser confocal measuring system. As a major contributor to the overall measurement uncertainty of the stylus profilometer, the repeatability, expressed as two standard deviations from five repeats, is also stated for each measurement. Other factors, such cut-off wavelength and stylus tip radius may have nanometer-level contribution to the overall uncertainty [42,43,44]. The measurement errors of the laser confocal system in Table 4 are plotted as the error curve in Figure 9.

For very smooth surfaces with *Ra* values less than 0.4 μm, the laser confocal system shows unpredictable errors, due to two typical inherent disadvantages of confocal technology namely the spot size limit and the background noise. The laser spot size limits the lateral resolution of the proposed measuring system which cannot resolve surface feature less than 1 µm. The background noise of the laser confocal sensor is mainly caused by laser power saturation, stray light and scattering effect at sharp edges [45]. 

In Figure 9, unique markers represent the different data of grinding, milling and turning for reference. Unfortunately, in practical measurement, machining patterns and Key Performance Indicators (KPIs) are sometimes unknown. In this study, therefore, a combined linear function was provided for error correction.

Accommodating a fixed intercept value will cause a notable relative error when the *Ra* value is very small. In this case, a zero-pass linear function *y =* 0.041*x* is forced to fit the measurement errors, as shown in Figure 9. The coefficient of determination R^2^ value, as usual, indicates how well the data points agree with the fitted line. The high R^2^ value of 0.9459 suggests that the data set can be regressed linearly.

**Figure 10:**
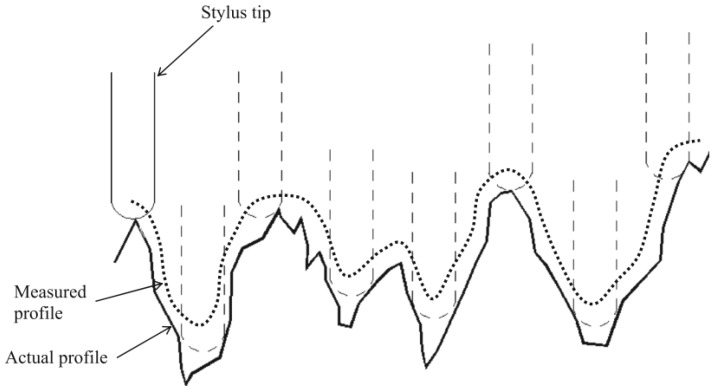
Actual and measured profiles using stylus method [46].

As observed in Figure 9, the measurement error increases linearly with increasing roughness *Ra*. This might be caused by two factors. Firstly, rough surfaces usually have sharper edges, defects and other small surface imperfections than smooth surfaces. These surface features can scatter the laser light away from the sensor objective and can lead to larger errors and more non-measured data points. Secondly, the stylus tip acts as a mechanical filter to reduce the peak-to-valley distance in surface profile (see Figure 10). This mechanical filter has more significant effect on the surface with higher roughness due to more small and narrow surface features.

**Figure 11:**
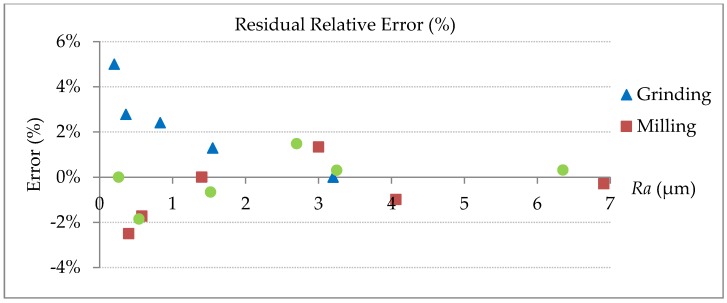
Residual relative errors after linear error compensation.

After linear correction, the residual relative errors can be calculated as the results shown in Figure 11. It can be seen that most of measurement relative errors can be controlled within 5%, regardless of the machining process. It is also shown that for small *Ra* values, the relative error is comparatively large, which means random errors contributed by spot size and noises are more influential for smooth surfaces. In this study, therefore, 0.2 μm has been observed to be the lower limit of the measurable *Ra* values due to the inherent limitations of laser confocal sensor.

## 5. Conclusions and Future Work

In this paper, a non-contact and in-situ surface topography measurement system has been developed using laser confocal technology. The objective of this study is to explore roughness measurement technology which can be used in the manufacturing environment instead of the traditional desktop measurement in laboratories. A laser confocal sensor has been integrated with a precision 2-axis linear stage and robot arm. Experimental data shows that the proposed system is able to measure surfaces with *Ra* from 0.2–7 μm, which covers a common range of milling, turning and grinding. In this range with linear error compensation, measurement relative errors can be controlled within 5%. From the above sections, the proposed laser confocal measuring system features the following attributes:

(1) High accuracy down to 0.2 μm *Ra* for roughness measurement, validated by a high-accuracy stylus profilometer,
(2) Non-contact measurement that prevent possible contamination and damage to sample surface,
(3) Compact design that can be integrated with a robot or other motion system for in-situ measurement,
(4) 3-Stage motion control that is able to minimize the vibration caused by robot and positioning motion mechanisms, and
(5) Low-cost design compared to the desktop system which consists of stylus or optical profilometer.

Based on the current measurement results and conclusion, we will focus on the following areas in the future work:

(1) Fabricate more roughness specimens with *Ra* in the range of 3–6 µm and validate the linear correction factor.
(2) Measure more roughness parameters such as *Rz* and *Rdq* which are more sensitive to profile peaks and valleys.
(3) Systematically investigate the optical noise and measurement error from the laser confocal sensor when measuring surface peaks and valleys.
(4) Systematically evaluate the uncertainty and repeatability of the proposed measuring system.

## Figures and Tables

**Table 1:** *Ra* values of the precision roughness reference specimen.

	1	2	3	4	5	Mean	Std. Dev.
*Ra* (µm)	3.11	3.03	3.01	3.14	3.08	3.07	0.05

**Table 2:** Stitching errors of the laser confocal profile.

	1	2	3	4	5	Mean	Std. Dev.
MSE (µm)	0.034	0.023	0.022	0.027	0.016	0.024	0.010

**Table 3:** Measurement results of the depth measurement standards.

	Nominal Value (µm)Instrument	5	10	15	20	25	30
Instrument							
Stylus (µm)		5.09 ± 0.02	9.95 ± 0.01	15.10 ± 0.02	19.98 ± 0.01	24.62 ± 0.05	29.99 ± 0.01
Confocal (µm)		5.24 ± 0.04	10.12 ± 0.05	15.21 ± 0.04	20.15 ± 0.11	24.78 ± 0.07	30.22 ± 0.05

**Table 4:** *Ra* of different surfaces measured using laser confocal system and stylus profilometer.

Machining	Stylus Profilometer Measured *Ra* (µm) (Reference)	Laser Confocal System Measured *Ra* (µm) (To Be Evaluated)	Error (µm)
**Grinding**	0.20 ± 0.01	0.22	0.02
	0.36 ± 0.01	0.38	0.02
	0.83 ± 0.01	0.88	0.05
	1.55 ± 0.02	1.63	0.08
	3.20 ± 0.04	3.33	0.13
**Milling**	0.40 ± 0.00	0.41	0.01
	0.58 ± 0.01	0.59	0.01
	1.40 ± 0.00	1.46	0.06
	3.00 ± 0.02	3.16	0.16
	4.06 ± 0.03	4.19	0.13
	6.91 ± 0.02	7.17	0.26
**Turning**	0.26 ± 0.01	0.27	0.01
	0.54 ± 0.01	0.55	0.01
	1.52 ± 0.02	1.57	0.05
	2.70 ± 0.02	2.85	0.15
	3.25 ± 0.04	3.39	0.14
	6.35 ± 0.02	6.63	0.28
